# Toxic Effects of Gemcitabine and Paclitaxel Combination: Chemotherapy Drugs Exposure in Zebrafish

**DOI:** 10.3390/toxics11060544

**Published:** 2023-06-20

**Authors:** Claudio D’Iglio, Sergio Famulari, Fabiano Capparucci, Claudio Gervasi, Salvatore Cuzzocrea, Nunziacarla Spanò, Davide Di Paola

**Affiliations:** 1 Department of Chemical, Biological, Pharmaceutical, and Environmental Science, University of Messina, 98166 Messina, Italy; cladiglio@unime.it (C.D.); serfamulari@unime.it (S.F.); fcapparucci@unime.it (F.C.); claudio.gervasi@unime.it (C.G.); dipaolad@unime.it (D.D.P.); 2 Department of Pharmacological and Physiological Science, Saint Louis University School of Medicine, 1402 S. Grand Blvd., St. Louis, MO 63104, USA

**Keywords:** gemcitabine, paclitaxel, zebrafish, chemical mixture, ROS

## Abstract

Pharmaceuticals are widely recognized as potentially hazardous to aquatic ecosystems. In the last two decades, the constant intake of biologically active chemicals used in human healthcare has been related to the growing release of these agents into natural environments. As reported by several studies, various pharmaceuticals have been detected, mainly in surface water (seas, lakes, and rivers), but also in groundwater and drinking water. Moreover, these contaminants and their metabolites can show biological activity even at very low concentrations. This study aimed to evaluate the developmental toxicity of exposure to the chemotherapy drugs gemcitabine and paclitaxel in aquatic environments. Zebrafish (*Danio rerio*) embryos were exposed to doses of gemcitabine 15 μM in combination with paclitaxel 1 μM from 0 to 96 h post-fertilization (hpf) using a fish embryo toxicity test (FET). This study highlights that both gemcitabine and paclitaxel exposure at single non-toxic concentrations affected survival and hatching rate, morphology score, and body length after exposure in combination. Additionally, exposure significantly disturbed the antioxidant defense system and increased ROS in zebrafish larvae. Gemcitabine and paclitaxel exposure caused changes in the expression of inflammation-related, endoplasmic reticulum stress-related (ERS), and autophagy-related genes. Taken together, our findings underline that gemcitabine and paclitaxel increase developmental toxicity in zebrafish embryos in a time-dependent manner.

## 1. Introduction

Gemcitabine (29,29-difluoro 29-deoxycytidine) is an analog of cytosine arabinoside (Ara-C) with many pharmacological properties and distinctive antitumor activity [1]. Gemcitabine’s (GEM) ability to slow the growth of human tumors has been demonstrated in a variety of hematological and solid cancer cell lines. Additionally, its in vivo activity has been demonstrated in murine solid tumors and human xenografts in nude mice [2]. In line with these preclinical studies, gemcitabine has shown important clinical activity. Currently, it is reported as a single compound in therapy for metastatic pancreatic cancer [3] and as association therapy in bladder cancer [4], non-small cell lung cancer [5], and breast cancer [6]. GEM has also been proven to be effective in other tumors, such as mesothelioma, ovarian cancer, and head and neck cancers [7]. Although GEM’s chemotherapic activities have been widely characterized, its effects on developmental-induced toxicity have not been elucidated. Paclitaxel, (1S,2S,3R,4S,7R,9S,10S,12R,15S)-4,12-diacetoxy-15-{[(2R,3S)-(benzoilammine)-2-hydroxy-3-fenilpropanoil]oxy}-1,9-diidroxy-10,14,17,17-tetrametil-11-oxy-6-oxatetracycle [11.3.1.0~3,10~.0~4,7~]eptadec-13-en-2-il benzoate) dissolved in cremophor EL and ethanol, is an antineoplastic medication that is commonly used to treat ovarian, breast, lung, and cervical cancers, as well as a variety of other head and neck neoplasms [8,9,10,11]. Paclitaxel (PAX) inhibits tumor cell proliferation by inducing tubulin polymerization, which prevents the formation of the mitotic spindle, causing proliferating cells to enter the G2/M phase of the cell cycle, eventually leading to tumor cell death via apoptosis or phagocytosis [12,13]. Numerous studies have shown therapeutic advantages in the use of GEM and PAX combinations in treating certain types of carcinomas [14,15]. The massive consumption of chemotherapic drugs is the main cause of the release of these compounds into aquatic environments. Wastewater treatment plants, hospital and industrial discharges, animal farming, and aquaculture facilities are the most significant pathways. In addition to freshwater locations such as lakes, rivers, groundwaters, and estuaries, recent studies also showed these substances in coastal areas [16]. Crustaceans, mollusks, and fishes were shown to accumulate pharmaceuticals under measured laboratory conditions [17], and various substances have been shown in wild marine and freshwater species, including mollusks (*Cassostrea* spp., *Mytilus* spp., *Elliptio* spp., *Dreissena* spp.), crustaceans (*Gammarus* spp.), macroalgae (*Laminaria* spp., *Saccharina* spp.), and fishes in different ecological categories (*Platichthys flesus*, *Liza aurata*, *Hemiculter leucisculus*, *Pleuronichthys verticalis*, *Carassius auratus*) [18,19]. These results highlight the emergent threat of pharmaceuticals to aquatic ecosystems and the importance of investigating their potentially harmful effects on aquatic species.

Zebrafish (*Danio rerio*) is a sensitive, reliable, and economic model extensively employed to determine the level of toxicity of various substances [20]. Zebrafish are easy to handle and are small in size, with visible embryological phases and high fecundity, and undergo rapid organogenesis and embryogenesis in vitro [21]. In fact, they show similarities at the molecular and physiological levels with humans in the respiratory system, cardiovascular system, and immune system [22,23,24]. Therefore, good correspondence with mammalian toxicity has been shown [25]. Zebrafish is employed as a universal model to evaluate the mechanism of action and toxicity during the early stages of embryonic development [26]. A previously published study demonstrated that GEM exposure at 25 μM is associated with congenital malformations and decreased survival rate [27] in zebrafish larvae. Additionally, several papers showed that GEM induced oxidative stress, skin cell senescence, cardiovascular, neuro- and hepatotoxicity, and embryonic toxicity [28]. However, the cellular effects of GEM and its mechanism of developmental toxicity are still poorly understood. Previous studies confirmed that ROS, the release of inflammatory cytokines, and gene toxicity are associated with developmental toxicity [27].

Inflammation is an adaptive response to guarantee that excessive stimuli are removed from the body and a healing process for rebuilding damaged tissue. However, chronic and persistent inflammation is detrimental [29]. Indeed, endoplasmic reticulum stress (ERS) and increasing ROS amounts are responsible for inflammation and autophagic cell death, which lead to the pathogenesis of a variety of diseases [30]. PAX’s capacity to trigger toxic events in various experimental models has been widely elucidated [31,32,33]. According to a recent study, the PAX-induced cell death process in AGS cells includes mitotic catastrophe, autophagy, and apoptosis. *Caspase-3*, *Caspase-9*, and PARP were all activated by PAX, which caused apoptosis. Additionally, the fact that PAX administration resulted in a large rise in the autophagy marker LC3B-II, along with *Atg5*, class III PI3K, and *beclin-1*, and a decrease in *p62* levels, confirmed that this anti-cancer medication triggered autophagy [34]. Moreover, previous studies demonstrated that PAX is able to cause neurotoxicity in zebrafish [35]. Exposure to PAX has also been shown to induce sensory axon degeneration and loss of touch response in a zebrafish model [36]. In the present study, we evaluated the detrimental effects of exposure to GEM in combination with PAX on malformation, oxidative stress, inflammatory response, and autophagy in zebrafish embryos. The regulation of expression of genes related to inflammation (*tgfb* and *cox2*), ERS (*hspa5*, *chop*, *ire1*, *xbp1s*, and *atf6*), and autophagy (*lc3*, *beclin1*, and *atg3*) pathways were explored.

## 2. Materials and Methods

### 2.1. Selection of Concentrations

We based our choice of non-toxic concentrations of GEM and PAX on environmental concentrations shown in the literature. GEM was reported to be detected in water at a range of 39.3 ng/ L^−1^ to 52 ng L^−1^ and PAX at a concentration between 1.40 mg L^−1^ and 2.19 mg L^−1^ [37]. Based on these ranges, we decided to use concentrations slightly lower than the environmental ones in order to have individually harmless doses of the drugs. A preliminary experiment was conducted in which three concentrations of GEM (15 μM, 25 μM, 35 μM) and three of PAX (0.5 μM, 1 μM, 2 μM) were selected. FET was then performed, and zebrafish embryos were exposed to these concentrations up to 96 hpf. From the results obtained on mortality and hatching rates, the highest single non-toxic concentrations were identified and were subsequently used for this study (Figure S1).

### 2.2. Solutions Preparation

GEM (Gemcitabine) 100 mg/mL and PAX (Paclitaxel) 6 mg/mL (30 mg/5 mL) were purchased (Accord Healthcare Italia S.r.l, city, Milan, Italy). The solutions were diluted in embryo medium, obtaining two different concentrations (GEM 15 μM, PAX 1 μM) and one concentration containing the mixture (GEM 15 μM + PAX 1 μM) in 24 wells (2 mL each) (Labsolute, Th. Geyer GmbH & Co. KG, 4–6 71272 Renningen, Germany), 1 for each solution and 1 plate with negative control (untreated), as previously shown [38].

### 2.3. Zebrafish Maintenance and Breeding

For the production of embryos, wild-type (WT) adult zebrafish (6 months old) were employed. Zebrafish were raised in the fish facility of the Department of Veterinary Sciences, University of Messina, Italy’s Center for Experimental Fish Pathology (Centro di Ittiopatologia Sperimentale della Sicilia, CISS). The fish were fed 3% of body weight (BW) of both dry and live food twice daily. Mature males and females were mated in a 2:1 ratio for successful reproduction. The following day, eggs were gathered and bleached, and non-fertilized eggs were discarded. Only blastula-stage embryos were used for this study.

### 2.4. Zebrafish Embryo Toxicity (ZFET) Assay

In accordance with OECD guidelines (OECD, Test No. 236: Fish Embryo Acute Toxicity (FET) test), the toxicity of GEM and PAX solutions was determined [39]. GEM (15 μM) and PAX (1 μM) were prepared using embryo medium (15 mM NaCl, 0.5 mM KCl, 1 mM CaCl_2_, 1 mM MgSO_4_, 0.15 mM KH_2_PO_4_, 0.05 mM Na_2_HPO_4_, 0.7 mM NaHCO_3,_ pH 7.3) and placed into a 24-well plate (1 embryo per well). Fertilized eggs were transferred into 24-well plates with test solutions (n = 24 in each plate for each replicate of an experimental group) and incubated at 26 °C at a 14:10 h day/night light regime. The experimental groups were divided as follows. One group was exposed to a solution of GEM 15 μM, one was exposed to a solution of PAX 1 μM, one was exposed to the mixture of GEM 15 μM + PAX 1 μM, and one was the control group (untreated). The experiment was repeated four times. The entire mortality and developmental abnormalities of embryos and larvae were observed and recorded at 24, 48, 72, and 96 hpf [40]. Coagulation, lack of somites, non-detachment of the tail, and no heartbeat were considered the lethal endpoint. Furthermore, the occurrence of defects in embryos during development was evaluated as a teratogenic endpoint. In addition, the percentage of hatchability and mortality were estimated. A stereo microscope was used to capture images and movies (Leica M205 C). Every 24 h, four separate endpoints were checked for any malformations.

(a) Embryo coagulation—This can occur within a few hours after exposure and indicates a generic acute toxic effect.
(b) Lack of somite formation—Twelve hours after fertilization, the somite should be visible; if not, the embryo will not continue to develop.
(c) Non-detachment of the tail—At 24 h after fertilization, the tail of the yolk sac can be seen detaching, indicating that the embryo is growing normally.
(d) Absence of heartbeat—The heartbeat can be easily felt 30 h after fertilization; its absence denotes the death of the embryo. Embryo coagulation and absence of heartbeat were focused on, both as endpoints of mortality.

### 2.5. Morphology Score and Body Lengths

The degree of the developmental toxicity effects observed in numerous organ systems (body shape, head, brain, somites, notochord, swim bladder, yolk sac, tail, fins, and pharyngeal arches/jaws) of zebrafish larvae (n = 20 for each group) after exposure to GEM + PAX was assessed at 96 hpf. Our study’s morphological scoring standards were previously described [41]. Using Image-Pro Plus software (Media Cybernetics, Bethesda, MD, USA), the length of each zebrafish larva was measured at 96 hpf WE along the body axis from the anterior-most region of the head to the tip of the tail.

### 2.6. Measurement of ROS Generation

Using 20, 70 di-chlorodihydrofluoresceindiacetate DCF-DA (Thermo Fisher Scientific Inc., Rome, Italy, ROS production in zebrafish larvae was assessed at 96 hpf. The larvae (n = 20 for each group) were incubated for 1 h in the dark at 28 °C in a solution of 20 mg/mL^−1^ DCF-DA. The larvae were then rinsed in new fish water. Finally, the larvae were observed using a fluorescent microscope (Olympus, Tokyo, Japan), and the fluorescence intensity was measured using the ImageJ program.

### 2.7. Total RNA Extraction and RT-PCR

First, a 4 °C pre-chill was applied to the centrifuge machine. Following several rounds of washing with sterile embryo medium (n = 20 per experimental group), embryos were carefully dried without harming them. After that, 500 μL of trizol was extracted and uniformly homogenized. Then, an additional 500 μL of trizol was added and thoroughly mixed. The samples were incubated for 15 min at 4 °C. Then, 200 μL of chloroform was added, vortexed vigorously for 15 s, and incubated for 2 to 3 min at room temperature. The samples underwent a 15 min, 12,000× *g* centrifugation at 4 °C. Next, the top aqueous phase was carefully transferred into a new tube, the same volume of 2-propanol was added, and it was incubated for 10 min on ice in order to precipitate the RNA. Samples were once more centrifuged for 30 min at 4 °C at a 12,000× *g* speed. The final steps included completely removing the supernatant, washing the RNA pellet on the side or bottom of the tube with 75% ethanol, and centrifuging it at 12,000× *g* for 5 min at 4 °C. The pellet underwent complete air-drying at room temperature. Following that, the pellet was resuspended in 50 μL of nuclease-free water and kept at −80 °C. Nanodrop was used to quantify the RNA (260/280), and a 1% agarose gel was used to verify the integrity of the isolated RNA. A Thermo Scientific cDNA synthesis kit (#K1622) was used to create the cDNA, and the instructions included with the kit were followed exactly. Then, after being normalized to the reference gene -actin, relative gene transcription levels were ascertained. The primer oligomers for the target genes are indicated in Table 1. The Design and Analysis software (v1.5.2, Applied Biosystems, Thermo Fischer Scientific) was used to analyze the melt curve and calculate the relative fold change using ΔΔct method in comparison to the control group.

### 2.8. Statistical Analyses

Data were presented as the mean ± standard error (SE). Significant differences between groups were determined using a two-way analysis of variance (ANOVA) and Bonferroni’s test. Statistical differences were considered significant at *** *p* < 0.0001.

## 3. Results

### 3.1. Mortality, Hatching Rate, and Malformations

**Figure 1:**
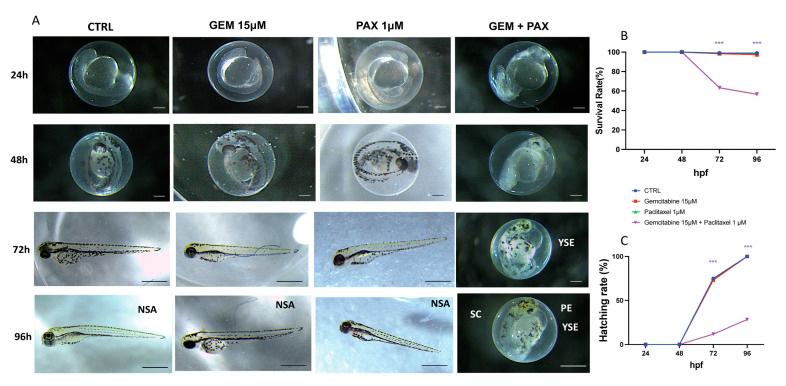
Developmental toxicity effect of GEM and PAX in zebrafish embryos. (**A**) Phenotypes of zebrafish embryos exposed to GEM and PAX from 24 to 96 hpf. (**B**) Mortality rate in zebrafish embryos exposed to GEM and PAX from 24 to 96 hpf. (**C**) Hatching rate in zebrafish embryos exposed to GEM and PAX from 24 to 96 hpf. *** *p* < 0.001 versus CTRL. (NSA) no showed abnormalities; (YSE) yolk sac edema; (SC) spinal cord teratogenesis; (PE) pericardial edema; scalebar 500 µm.

**Figure 2:**
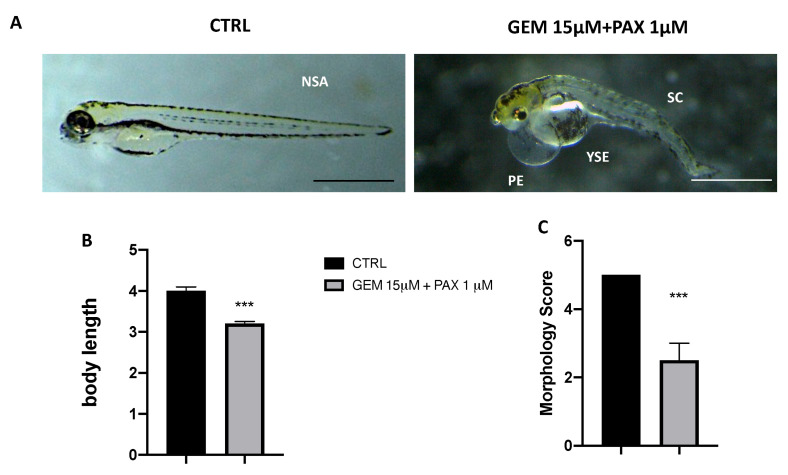
Effects of GEM and PAX on morphological changes in zebrafish larvae at 96 hpf. (**A**) Representative lateral views, body length (**B**), and morphological scoring (**C**) of zebrafish larvae treated with GEM 15 µM andPAX 1 µM. *** *p* < 0.001 versus CTRL. (NSA) no showed abnormalities; (YSE) yolk sac edema; (SC) spinal cord teratogenesis; (PE) pericardial edema; scalebar 500 µm.

The mortality rates of zebrafish embryos exposed to GEM 15 µM, PAX 1 µM, and a combination of GEM 15 µM and PAX 1 µM for 24–96 hpf are shown in Figure 1A. Single concentrations of GEM and PAX showed no significant difference in mortality from 24 hpf to 96 hpf. However, a high mortality rate was found in the GEM 15 µM + PAX 1 µM exposure group at 72 hpf and 96 hpf, respectively. The hatching rates are shown in Figure 1B. Significantly decreased hatching rates were observed at 48–96 hpf for the combined dose of GEM 15 µM and PAX 1 µM. These data indicated a remarkable decrease in the hatching rate induced by GEM and PAX (Figure 1B). The phenotypic defects caused by GEM and PAX from 24 hpf to 96 hpf are shown in Figure 1A and Figure 2A. An apparent delay in hatching was also found in the combination dose of GEM and PAX. In the GEM 15 μM and PAX 1 µM single exposure groups, no statistically relevant malformation was observed (Table 2). When GEM was combined with PAX, severe developmental abnormalities became very noticeable, including severe yolk sac edema, spinal cord teratogenesis, and pericardial edema. These growth defects may be caused by abnormal cell death.

### 3.2. Body Length in GEM-PAX Exposed Zebrafish

The degree of development was determined by measuring changes in the larvae body lengths. The body lengths of the larvae in the GEM 15 µM and PAX 1 µM treated group were noticeably smaller compared to the control group. (Figure 2B). Our results showed that GEM and PAX together significantly inhibited larval growth and development (Figure 2C).

### 3.3. ROS Measurement

**Figure 3:**
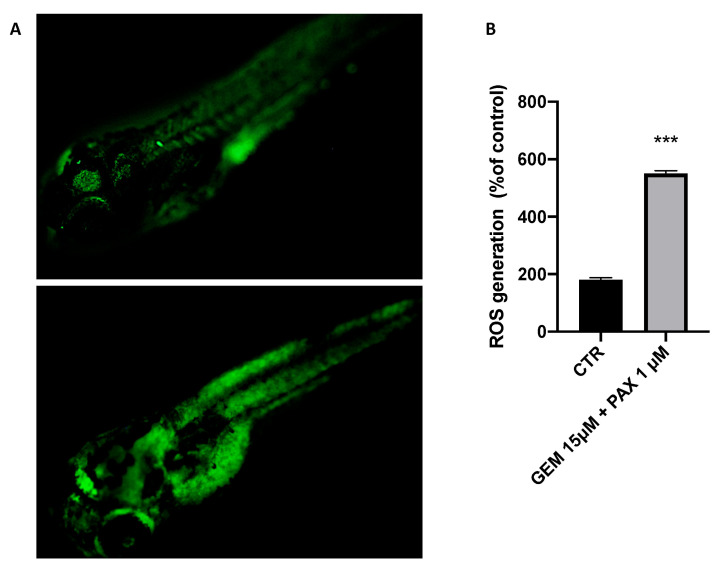
Effects of ROS production in zebrafish larvae at 96 hpf. (**A**) Fluorescence micrographs of ROS generation in zebrafish larvae. (**B**) Quantitative analysis of ROS generation using the Image J program. *** at *p* < 0.0001 against CTRL.

As shown in Figure 3A, GEM + PAX exposure increased the generation of ROS in the treated zebrafish larvae group compared with the control group.

### 3.4. Gene Expression

**Figure 4:**
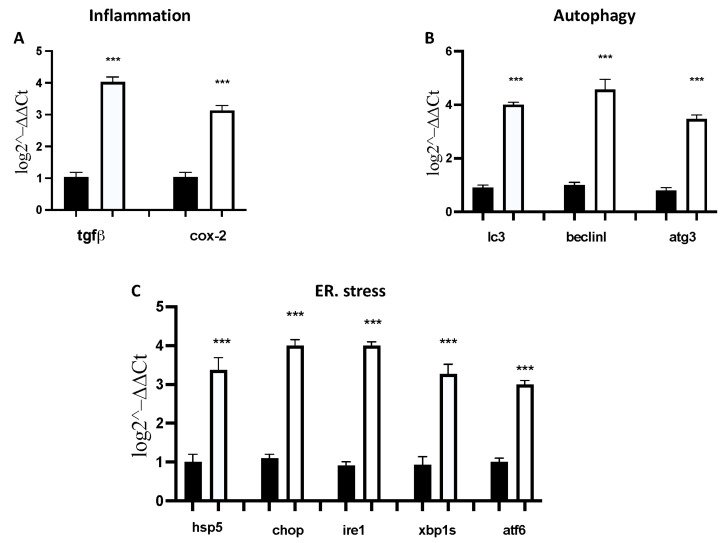
The GEM 15 μM + PAX 1 μM exposure effects on inflammation (**A**), autophagy (**B**), and ER stress (**C**) related genes on zebrafish embryos. The results are expressed as the mean of four independent experiment data. The expression levels of mRNA are represented as the fold change from the CTRL group. *** *p* < 0.001.

An RT-PCR experiment was performed at 96 hpf to evaluate the probable mechanism underlying the developmental toxicity of GEM + PAX exposure. When exposed by 96 hpf, the GEM + PAX-treated group demonstrated up-regulation of two inflammation-related genes (*tgfb* and *cox2*), five ERS-related genes (*hspa5*, *chop*, *ire1*, *xbp1s*, *and atf6*), and three autophagy-related genes (*lc3*, *beclin1*, and *atg3*), as compared to the control group. (Figure 4).

## 4. Discussion

The environmental effects of pharmaceuticals in aquatic ecosystems are now considered a critical problem with deleterious outcomes and global-scale distribution. The rising trend of drug intake and release in aquatic environments highlights the necessity for special national and international actions to address this issue. Several scientific studies have shown the onset of significant effects on early cellular, biochemical, and molecular responses in aquatic species exposed to environmental concentrations of pharmaceuticals [42,43,44]. Moreover, pharmaceuticals are a class of contaminants of emerging concern, and one of their characteristics is that they can generate a biological response even at very low concentrations [45]. For the aquatic environment, genotoxic effects are predicted to be a serious issue [46], but unfortunately, the literature on the genotoxicity of cytostatic drugs is quite poor. To date, investigations have suggested that the studied cytostatic medicines’ ambient concentrations are below the levels required to generate genotoxic effects. For example, doxorubicin has the lowest genotoxic effect (74 µg/L^−1^), while the maximum concentration found in effluent wastewater (0.042 µg/L^−1^) was three orders of magnitude lower in the worst-case scenario [37]. A previous study confirmed that no apparent toxicity was found in embryos after treatment with GEM at 15 μM, and evident developmental toxicity was observed for exposure higher than 25 μM [27]. According to field studies over the years, PAX also does not appear to pose an alarming threat to aquatic organisms, given the environmental concentrations found [37]. The use of combinations of drugs in various therapies (especially those for cancer treatment) does not exclude the possibility that these substances, which are individually harmless at these concentrations, may lead to a clear toxic effect in combination with each other and, therefore, be a threat to the environment. Single doses of GEM and PAX, 15 µM and 1 µM, respectively, showed no relevant changes in the survival rate and hatching of larvae exposed up to 96 hpf. These data appear to be in agreement with previous studies. GEM 15 µM + PAX 1 µM exposure, on the contrary, showed three major types of developmental abnormalities: spinal cord teratogenesis, pericardial edema, and yolk sac edema. Our results demonstrated that exposure to GEM 15 µM and PAX 1 µM significantly affected mortality and caused malformation, affecting hatching rate and body length. These results were consistent with those of Benyumov et al. 2011 [27]. According to the results of previous studies [27,31,34], in order to evaluate a possible pathway of toxicity underlying the GEM/PAX exposure, we investigated the main pathways involved in developmental toxicity. Our data showed increased inflammation and generation of ROS. The mRNA level of the inflammatory mediators *tgfb* and *cox2* were found to be significantly increased, and the antioxidant defense system was found to be altered as well after GEM + PAX exposure. The up-regulation of the proinflammatory mediators and the persistence of ROS can induce ER damage [47], thus, we evaluated the ERS gene expression. ER is involved in the management of proteins functions such as synthesis, folding, and delivery [48]. In case of unfolded proteins accumulation, ER activates the unfolded protein response (UPR) to re-establish normal ER functions and to help the cells to adapt to environmental changes [49]. However, UPR can also induce cell death when ERS is prolonged and severe [50]. Three signaling pathways can activate UPR: the inositol-requiring enzyme 1 (IRE1)-X-box binding protein 1 (XBP1) pathway, the protein kinase RNA-activated-like ER kinase (PERK)-eukaryotic translation initiation factor 2 alpha (eIF2a) pathway, and the activating transcription factor 6 (ATF6) pathway [51]. *Hspa5* is a regulator of the ER homeostasis activated in case of accumulation of misfolded and unfolded proteins in ER [52]. *Chop* is a regulator of the apoptosis pathway activated by ERS [53], while *ire1* is responsible for the activation of the endoribonuclease activity that activates the *xbp1s* transcription factor. The mRNA expression of *hspa5*, *chop*, *ire1*, *xbp1s*, and *atf6* was found to be increased in zebrafish embryos, indicating that GEM 15 µM and PAX 1 µM co-exposure probably trigger ERS through the IRE1-XBP1 and ATF6 pathways. Several studies highlighted that autophagy is stimulated for cell survival after ERS [54]. However, persistent activation of this pathway by ERS leads to cell injury [50]. It has been reported that *atf6* indirectly regulates autophagy via *chop* and *xbp1* [30]. *Xbp1* is mediated by *ire1* and activates autophagy via *beclin1* [55]. *Beclin1* is involved in the initiation and nucleation of autophagy, while *lc3* and *atg3* are involved in the extension and closure of the autophagic membrane [30]. Our analysis also showed increased mRNA expressions of autophagy genes *beclin1*, *lc3*, and *atg3*.

## 5. Conclusions

Our experiments show that exposure to GEM and PAX at 15 μM and 1 μM concentrations, respectively, does not lead to statistically significant toxicity episodes in zebrafish larvae; however, their co-exposure at the same concentrations strongly affects the survival rate and delay in hatching and also causes growth and developmental aberrations, increasing inflammation, ROS production, ERS, and autophagy. Certainly, further studies are needed to investigate other possible biomarkers of toxicity in order to highlight the molecular mechanisms of toxicity.

## Figures and Tables

**Table 1:** Primers for real-time PCR.

Gene	Primer Orientation	Nucleotide Sequence
*β-actin*	forward	5′-AGAGCTATGAGCTGCCTGACG-3′
	reverse	5′-CCGCAAGATTCCATACCCA-3′
*tgfβ*	forward	5′-GAACTCGCTTTGTCTCCA-3′
	reverse	5′-TACAGTCGCAGTATAACCTCA-3′
*cox2*	forward	5′-ATCCTGTTGTCAAGGTCCCA-3′
	reverse	5′-CAAGGGTGCGGGTGTAAT-3′
*hspa5*	forward	5′-CAGATCTGGCCAAAATGCGG-3′
	reverse	5′-GGAACAAGTCCATGTTGAGC-3′
*chop*	forward	5′-CACAGACCCTGAATCAGAAG-3′
	reverse	5′-CCACGTGTCTTTTATCTCCC-3′
*ire1*	forward	5′-TGACGTGGTGGAAGTTGGTA-3′
	reverse	5′-ACGGATCACACATTGGGATGTT-3′
*xbp1s*	forward	5′-CAAAGGAGCAGGTTCAGGTAC-3′
	reverse	5′-GGAGATCAGACTCAGAGTCTG-3′
*atf6*	forward	5′-CTGTGGTGAAACCTCCACCT-3′
	reverse	5′-CATGGTGACCACAGGAGATG-3′
*lc3*	forward	5′-AAAGGAGGACATTTGAGCAG-3′
	reverse	5′-AATGTCTCCTGGGAAGCGTA-3′
*beclinl*	forward	5′-AGAGCATTGAGACAAAGCGTGAA-3′
	reverse	5′-TCTGCCAAGGCGGAAGTTATT-3′
*atg3*	forward	5′-GGCTGTTTGGATATGATGAG-3′
	reverse	5′-AGCAGGTGGAGGGAGATTAG-3′

**Table 2:** GEM and PAX effects on zebrafish larvae at 96 hpf.

		Malformations			Incidence (hpf)		
	SC	PE	YSE	24 h	48 h	72 h	96 h
CTRL	0	0	0	0	0	0	0
GEM 15 μM	<2%	0	0	0	0	0	<2%
PAX 1 µM	0	0	<3%	0	0	<3%	<3%
GEM 15 μM + PAX 1 µM	35%	40%	75%	3%	3%	70%	70%

Spinal cord teratogenesis (SC); Pericardial edema (PE); Yolk sac edema (YE).

## Data Availability

Data is contained within the article or supplementary material.

## Supplementary Materials

The following supporting information can be downloaded at: https://www.mdpi.com/article/10.3390/toxics11060544/s1, Figure S1: Survival rate and hatching of embryos exposed to different concentrations of GEM and PAX.
