# Supplementary material for: Toxic Effects of Gemcitabine and Paclitaxel Combination: Chemotherapy Drugs Exposure in Zebrafish
Source: Toxics. 2023 Jun 20;11(6):544. doi: 10.3390/toxics11060544 (PMC10301628; doi:10.3390/toxics11060544)
Supplement: Supplementary file 1 [file toxics-11-00544-s001.zip › toxics-2409822-supplementary.pdf]

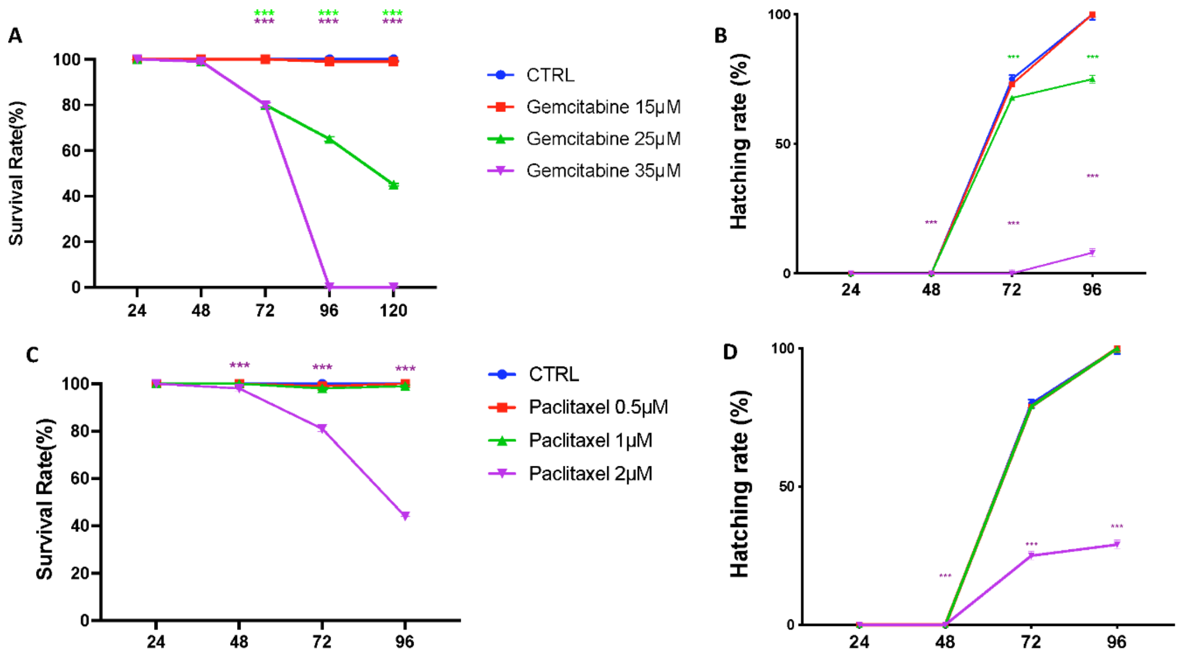

Figure S1. Survival rate and hatching of embryos exposed to different concentrations GEM and PAX. (**A**) Survival rate of GEM (gemcitabine) from 24 to 96hpf, (**B**) Hatching rate of GEM (gemcitabine) from 24 to 96hpf, (**C**) Survival rate of PAX (paclitaxel) from 24 to 96hpf, (**D**) Hatchingrate of PAX (paclitaxel) from 24 to 96hpf.
